# (*R*)-1-[(*S*)-(3-Cyano­thio­morpholino)carbon­yl]-2-methyl­propyl­aminium chloride dihydrate

**DOI:** 10.1107/S1600536809050624

**Published:** 2009-11-28

**Authors:** Pengfei Chen, Lele Liu, Junhai Xiao, Wu Zhong, Song Li

**Affiliations:** a Inner Mongolia Medical College, Hohhot 010059, People’s Republic of China; b Beijing Institute of Pharmacology and Toxicology, Beijing 100850, People’s Republic of China

## Abstract

In the title compound, C_10_H_18_N_3_OS^+^·Cl^−^·2H_2_O, the three C atoms of the isopropyl group are disordered and were refined using a split-site mode [occupancy ratio 0.53 (3):0.47 (3)]. In the crystal, the cations, anions and water mol­ecules are connected *via* O—H⋯O, O—H⋯Cl, N—H⋯Cl and N—H⋯O hydrogen bonding.

## Related literature

For *N*-substituted thio­morpholine derivatives as potentialdipeptidyl peptidase IV(DPP-IV) inhibitors, see: Engel *et al.* (2003 ▶). For their biological activity, see: Mu *et al.* (2006 ▶); Proost *et al.* (1998 ▶). For the synthesis, see: Li *et al.* (2007 ▶)
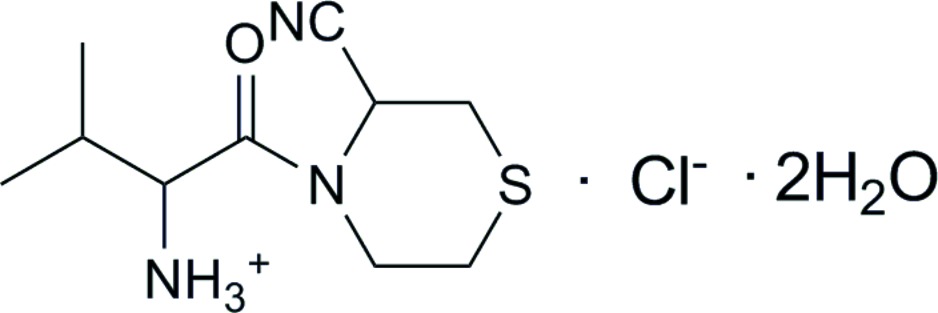


## Experimental

### 

#### Crystal data

C_10_H_18_N_3_OS^+^·Cl^−^·2H_2_O
*M* *_r_* = 299.82
Monoclinic, 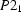

*a* = 9.6425 (19) Å
*b* = 6.8180 (14) Å
*c* = 12.082 (2) Å
β = 99.25 (3)°
*V* = 784.0 (3) Å^3^
*Z* = 2
Mo *K*α radiation
μ = 0.38 mm^−1^
*T* = 113 K
0.24 × 0.20 × 0.16 mm

#### Data collection

Rigaku Saturn CCD area-detector diffractometer
Absorption correction: multi-scan (*CrystalClear*; Rigaku/MSC, 2005 ▶) *T*_min_ = 0.914, *T*_max_ = 0.942
6464 measured reflections
3554 independent reflections
2987 reflections with *I* > 2σ(*I*)
*R*_int_ = 0.028

#### Refinement

*R*[*F* ^2^ > 2σ(*F* ^2^)] = 0.027
*wR*(*F* ^2^) = 0.066
*S* = 1.00
3554 reflections
216 parameters
49 restraints
H atoms treated by a mixture of independent and constrained refinement
Δρ_max_ = 0.20 e Å^−3^
Δρ_min_ = −0.35 e Å^−3^
Absolute structure: Flack (1983 ▶), 1544 Friedel pairs
Flack parameter: −0.03 (4)

### 

Data collection: *CrystalClear* (Rigaku/MSC, 2005 ▶); cell refinement: *CrystalClear*; data reduction: *CrystalClear*; program(s) used to solve structure: *SHELXS97* (Sheldrick, 2008 ▶); program(s) used to refine structure: *SHELXL97* (Sheldrick, 2008 ▶); molecular graphics: *SHELXTL* (Sheldrick, 2008 ▶); software used to prepare material for publication: *SHELXTL*.

## Supplementary Material

Crystal structure: contains datablocks I, global. DOI: 10.1107/S1600536809050624/nc2164sup1.cif

Structure factors: contains datablocks I. DOI: 10.1107/S1600536809050624/nc2164Isup2.hkl

Additional supplementary materials:  crystallographic information; 3D view; checkCIF report

## Figures and Tables

**Table 1:** Hydrogen-bond geometry (Å, °)

*D*—H⋯*A*	*D*—H	H⋯*A*	*D*⋯*A*	*D*—H⋯*A*
N3—H3*A*⋯O2^i^	0.96 (2)	1.89 (2)	2.838 (2)	169.3 (15)
N3—H3*B*⋯Cl1	0.97 (2)	2.37 (2)	3.3178 (16)	165.3 (15)
N3—H3*C*⋯O1^ii^	0.807 (18)	2.232 (18)	2.7891 (17)	126.6 (17)
N3—H3*C*⋯Cl1^ii^	0.807 (18)	2.599 (19)	3.2732 (16)	142.0 (16)
O2—H2*A*⋯Cl1^i^	0.85 (2)	2.37 (3)	3.2176 (15)	171 (2)
O2—H2*B*⋯O3	0.92 (2)	1.81 (2)	2.7186 (19)	171 (2)
O3—H3*F*⋯Cl1	0.82 (2)	2.31 (2)	3.1332 (15)	179 (2)
O3—H3*G*⋯O2^i^	0.86 (3)	2.02 (3)	2.873 (2)	173 (2)

Symmetry codes: (i) 
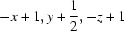
; (ii) 
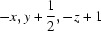
.

**Fig. 1.**
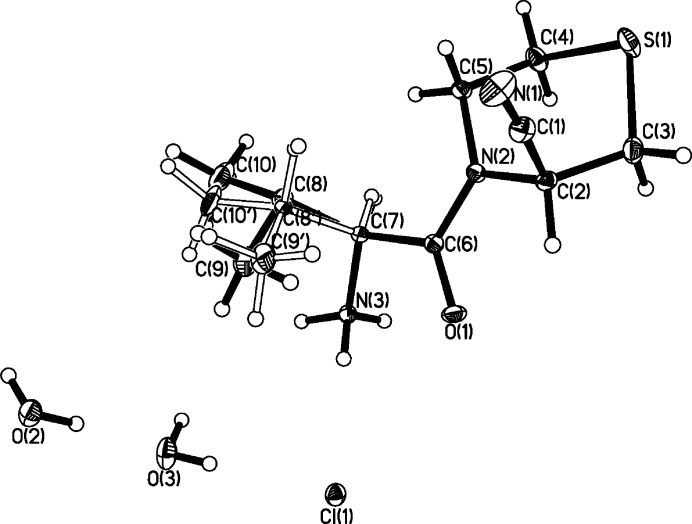
Crystal structure of the title compound with the atomic numbering scheme.Displacement ellipsoids are shown at the 50% probability level.

**Fig. 2.**
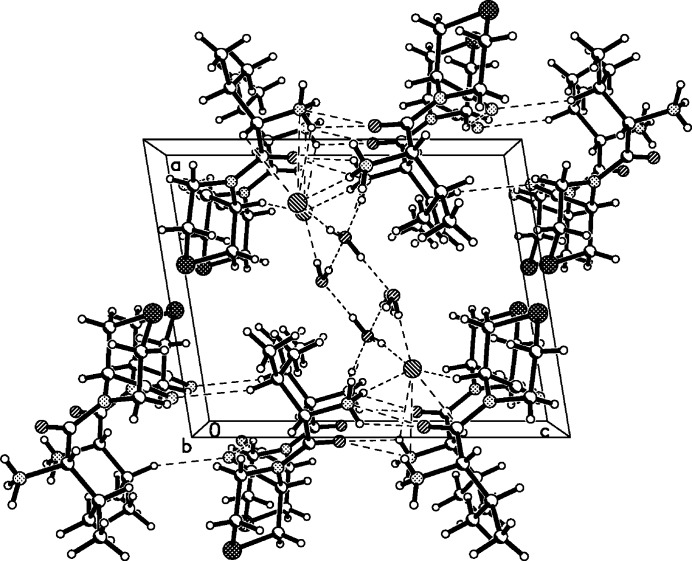
Crystal structure of the title compound with view along the b axis. Hydrogen bonding is shown as dashed lines.

## supplementary crystallographic information

### Comment

N-substituted thiomorpholine derivatives are considered as a potential dipeptidyl peptidase IV(DPP-IV) (Engel *et al.*, 2003) inhibitor. These compounds present various biological properties, for instance, effectively ameliorate hyperglycemia,hyperlipidemia and significantly increase β-cell mass and improve islet architecture (Mu *et al.*, 2006). In Addition, DPP-4 inhibitors display a potential anti-HIV-1 activity (Proost *et al.*, 1998). The crystal structure of the title compound was analyzed by X-ray diffraction to investigate its structure-activity relationships.

In the crystal structure molecule of the title compound (Fig.1) the cations and anions are linked *via* intermolecular N—H···Cl interactions. They are additionally connected to the crystal water molecules by N—H···O and O—H···Cl interactions (Fig. 2 and Table 1). The water molecules are linked by strong O—H···O hydrogen bonding into zigzag-chains that elongate in the direction of the crystallographic *c* axis (Fig. 2).

### Experimental

The title compound was synthesized according the reported procedure of Li *et al.*(2007). Colorless single crystals were obtained by slow evaporation of a solution in acetone.

### Refinement

The C—H H atoms were placed in ideal positions and were refined using a riding model. with C—H=0.98–1.00Å and *U*_iso_(H)=1.2*U*_eq_(C) (1.5 for methyl H atoms). The N—H and O—H H atoms were refined with varying coordinates isotropic with *U*_iso_(H) = 1.2*U*_eq_(N) =1.5*U*_eq_(O). The isopropyl group is disordered and were refined using a split model with restrained distances and s.o.f. of 0.54 (3) and 0.46 (3). The absolute structure was determined on the basis of 1535 Friedel pairs.

### Crystal data

**Table:**

C_10_H_18_N_3_OS^+^·Cl^−^·2H_2_O	*F*(000) = 320
*M**_r_* = 299.82	*D*_x_ = 1.270 Mg m^−^^3^
Monoclinic, *P*2_1_	Mo *K*α radiation, λ = 0.71073 Å
*a* = 9.6425 (19) Å	Cell parameters from 2714 reflections
*b* = 6.8180 (14) Å	θ = 3.0–27.9°
*c* = 12.082 (2) Å	µ = 0.38 mm^−^^1^
β = 99.25 (3)°	*T* = 113 K
*V* = 784.0 (3) Å^3^	Block, colorless
*Z* = 2	0.24 × 0.20 × 0.16 mm

### Data collection

**Table:**

Rigaku Saturn CCD area-detector diffractometer	3554 independent reflections
Radiation source: rotating anode	2987 reflections with *I* > 2σ(*I*)
confocal	*R*_int_ = 0.028
Detector resolution: 7.31 pixels mm^-1^	θ_max_ = 27.9°, θ_min_ = 1.7°
ω and φ scans	*h* = −12→12
Absorption correction: multi-scan (*CrystalClear*; Rigaku/MSC, 2005)	*k* = −8→8
*T*_min_ = 0.914, *T*_max_ = 0.942	*l* = −11→15
6464 measured reflections	

### Refinement

**Table:**

Refinement on *F*^2^	Secondary atom site location: difference Fourier map
Least-squares matrix: full	Hydrogen site location: inferred from neighbouring sites
*R*[*F*^2^ > 2σ(*F*^2^)] = 0.027	H atoms treated by a mixture of independent and constrained refinement
*wR*(*F*^2^) = 0.066	*w* = 1/[σ^2^(*F*_o_^2^) + (0.0336*P*)^2^] where *P* = (*F*_o_^2^ + 2*F*_c_^2^)/3
*S* = 1.00	(Δ/σ)_max_ = 0.001
3554 reflections	Δρ_max_ = 0.20 e Å^−^^3^
216 parameters	Δρ_min_ = −0.35 e Å^−^^3^
49 restraints	Absolute structure: Flack (1983), 1535 Friedel pairs
Primary atom site location: structure-invariant direct methods	Flack parameter: −0.03 (4)

### Special details

**Table:**

Geometry. All e.s.d.'s (except the e.s.d. in the dihedral angle between two l.s. planes) are estimated using the full covariance matrix. The cell e.s.d.'s are taken into account individually in the estimation of e.s.d.'s in distances, angles and torsion angles; correlations between e.s.d.'s in cell parameters are only used when they are defined by crystal symmetry. An approximate (isotropic) treatment of cell e.s.d.'s is used for estimating e.s.d.'s involving l.s. planes.
Refinement. Refinement of *F*^2^ against ALL reflections. The weighted *R*-factor *wR* and goodness of fit *S* are based on *F*^2^, conventional *R*-factors *R* are based on *F*, with *F* set to zero for negative *F*^2^. The threshold expression of *F*^2^ > σ(*F*^2^) is used only for calculating *R*-factors(gt) *etc*. and is not relevant to the choice of reflections for refinement. *R*-factors based on *F*^2^ are statistically about twice as large as those based on *F*, and *R*- factors based on ALL data will be even larger.

### Fractional atomic coordinates and isotropic or equivalent isotropic displacement parameters (Å^2^)

**Table:**

	*x*	*y*	*z*	*U*_iso_*/*U*_eq_	Occ. (<1)
S1	−0.41733 (4)	0.64231 (8)	0.03966 (4)	0.03201 (12)	
O1	−0.03965 (11)	0.5796 (2)	0.38313 (8)	0.0233 (3)	
N1	−0.12007 (18)	0.2338 (3)	0.05250 (14)	0.0364 (4)	
N2	−0.12711 (12)	0.6463 (2)	0.20239 (10)	0.0155 (3)	
N3	0.10799 (15)	0.8983 (2)	0.42585 (11)	0.0158 (3)	
H3A	0.186 (2)	0.986 (3)	0.4463 (14)	0.024*	
H3B	0.1398 (19)	0.784 (3)	0.4706 (15)	0.024*	
H3C	0.041 (2)	0.947 (3)	0.4483 (14)	0.024*	
C1	−0.16317 (19)	0.3355 (3)	0.11428 (15)	0.0232 (4)	
C2	−0.21136 (16)	0.4683 (3)	0.19675 (12)	0.0181 (3)	
H2	−0.1927	0.4031	0.2718	0.022*	
C3	−0.36913 (17)	0.5114 (3)	0.16917 (13)	0.0251 (4)	
H3D	−0.3975	0.5895	0.2309	0.030*	
H3E	−0.4214	0.3859	0.1648	0.030*	
C4	−0.29541 (17)	0.8435 (3)	0.07232 (15)	0.0253 (4)	
H4A	−0.3025	0.9325	0.0069	0.030*	
H4B	−0.3208	0.9191	0.1361	0.030*	
C5	−0.14514 (17)	0.7707 (3)	0.10221 (13)	0.0180 (3)	
H5A	−0.1198	0.6951	0.0384	0.022*	
H5B	−0.0809	0.8845	0.1156	0.022*	
C6	−0.04067 (15)	0.6840 (2)	0.30047 (12)	0.0154 (3)	
C7	0.06389 (15)	0.8528 (2)	0.30441 (11)	0.0148 (3)	
H7A	0.0150	0.9647	0.2691	0.018*	0.53 (3)
H7B	0.0196	0.9649	0.2656	0.018*	0.47 (3)
C8	0.1874 (9)	0.8031 (14)	0.2407 (11)	0.024 (3)	0.53 (3)
H8	0.1468	0.7550	0.1642	0.028*	0.53 (3)
C9	0.2793 (11)	0.641 (2)	0.3005 (11)	0.041 (2)	0.53 (3)
H9A	0.3459	0.5962	0.2526	0.062*	0.53 (3)
H9B	0.2200	0.5310	0.3161	0.062*	0.53 (3)
H9C	0.3311	0.6917	0.3711	0.062*	0.53 (3)
C10	0.2733 (12)	0.9874 (18)	0.2277 (11)	0.047 (2)	0.53 (3)
H10A	0.3165	1.0345	0.3019	0.070*	0.53 (3)
H10B	0.2116	1.0893	0.1897	0.070*	0.53 (3)
H10C	0.3470	0.9566	0.1832	0.070*	0.53 (3)
C8'	0.1900 (8)	0.7874 (14)	0.2504 (11)	0.019 (3)	0.47 (3)
H8'	0.1524	0.7688	0.1690	0.023*	0.47 (3)
C9'	0.2515 (12)	0.5906 (16)	0.2911 (13)	0.034 (2)	0.47 (3)
H9D	0.3310	0.5595	0.2528	0.052*	0.47 (3)
H9E	0.1795	0.4887	0.2746	0.052*	0.47 (3)
H9F	0.2839	0.5964	0.3722	0.052*	0.47 (3)
C10'	0.3015 (10)	0.9447 (18)	0.2549 (12)	0.036 (2)	0.47 (3)
H10D	0.3581	0.9474	0.3299	0.053*	0.47 (3)
H10E	0.2564	1.0726	0.2385	0.053*	0.47 (3)
H10F	0.3622	0.9161	0.1992	0.053*	0.47 (3)
Cl1	0.21552 (4)	0.56119 (6)	0.61863 (3)	0.01970 (9)	
O2	0.67759 (13)	0.6702 (2)	0.49074 (11)	0.0268 (3)	
H2A	0.703 (2)	0.768 (4)	0.4543 (18)	0.040*	
H2B	0.611 (2)	0.735 (4)	0.5235 (17)	0.040*	
O3	0.47478 (13)	0.8267 (2)	0.59628 (12)	0.0324 (3)	
H3F	0.407 (3)	0.756 (4)	0.6016 (18)	0.049*	
H3G	0.436 (2)	0.934 (4)	0.5706 (18)	0.049*	

### Atomic displacement parameters (Å^2^)

**Table:**

	*U*^11^	*U*^22^	*U*^33^	*U*^12^	*U*^13^	*U*^23^
S1	0.0217 (2)	0.0299 (3)	0.0392 (3)	−0.00703 (19)	−0.01085 (17)	0.0057 (2)
O1	0.0302 (6)	0.0248 (7)	0.0141 (5)	−0.0081 (5)	0.0005 (4)	0.0054 (5)
N1	0.0515 (11)	0.0204 (10)	0.0419 (10)	−0.0008 (8)	0.0216 (8)	−0.0005 (7)
N2	0.0165 (6)	0.0134 (7)	0.0157 (6)	−0.0021 (6)	−0.0002 (5)	0.0013 (5)
N3	0.0151 (6)	0.0182 (8)	0.0143 (6)	−0.0016 (6)	0.0031 (5)	−0.0042 (5)
C1	0.0285 (9)	0.0151 (9)	0.0258 (8)	−0.0034 (7)	0.0040 (7)	0.0033 (7)
C2	0.0219 (8)	0.0168 (9)	0.0157 (7)	−0.0055 (6)	0.0031 (6)	0.0016 (6)
C3	0.0200 (8)	0.0262 (11)	0.0292 (9)	−0.0059 (7)	0.0045 (6)	−0.0007 (7)
C4	0.0210 (8)	0.0209 (10)	0.0309 (9)	−0.0011 (7)	−0.0046 (7)	0.0055 (8)
C5	0.0194 (8)	0.0163 (9)	0.0173 (8)	−0.0031 (6)	−0.0002 (6)	0.0055 (6)
C6	0.0159 (7)	0.0161 (9)	0.0145 (7)	0.0012 (6)	0.0029 (5)	−0.0016 (6)
C7	0.0149 (7)	0.0175 (9)	0.0115 (7)	−0.0009 (6)	0.0008 (5)	0.0001 (6)
C8	0.019 (4)	0.035 (4)	0.019 (4)	0.008 (3)	0.007 (3)	0.003 (3)
C9	0.022 (3)	0.065 (5)	0.039 (3)	0.015 (3)	0.011 (3)	0.006 (4)
C10	0.043 (4)	0.064 (5)	0.038 (4)	−0.028 (3)	0.021 (3)	−0.007 (3)
C8'	0.015 (4)	0.035 (5)	0.008 (3)	−0.011 (3)	0.000 (3)	−0.003 (3)
C9'	0.024 (3)	0.040 (4)	0.042 (4)	0.018 (3)	0.013 (3)	0.009 (3)
C10'	0.028 (3)	0.049 (4)	0.036 (4)	−0.012 (3)	0.022 (3)	−0.011 (3)
Cl1	0.01873 (17)	0.0176 (2)	0.02289 (18)	−0.00188 (16)	0.00372 (13)	0.00047 (16)
O2	0.0250 (6)	0.0215 (8)	0.0359 (7)	0.0045 (6)	0.0106 (5)	0.0060 (6)
O3	0.0219 (7)	0.0244 (8)	0.0526 (9)	−0.0029 (6)	0.0118 (6)	0.0001 (7)

### Geometric parameters (Å, °)

**Table:**

S1—C3	1.7965 (17)	C7—H7A	0.9601
S1—C4	1.8085 (18)	C7—H7B	0.9601
O1—C6	1.2251 (18)	C8—C9	1.523 (8)
N1—C1	1.145 (2)	C8—C10	1.527 (7)
N2—C6	1.3594 (18)	C8—H8	1.0000
N2—C2	1.456 (2)	C9—H9A	0.9800
N2—C5	1.465 (2)	C9—H9B	0.9800
N3—C7	1.4931 (18)	C9—H9C	0.9800
N3—H3A	0.96 (2)	C10—H10A	0.9800
N3—H3B	0.97 (2)	C10—H10B	0.9800
N3—H3C	0.807 (18)	C10—H10C	0.9800
C1—C2	1.476 (2)	C8'—C10'	1.514 (7)
C2—C3	1.533 (2)	C8'—C9'	1.517 (8)
C2—H2	1.0000	C8'—H8'	1.0000
C3—H3D	0.9900	C9'—H9D	0.9800
C3—H3E	0.9900	C9'—H9E	0.9800
C4—C5	1.519 (2)	C9'—H9F	0.9800
C4—H4A	0.9900	C10'—H10D	0.9800
C4—H4B	0.9900	C10'—H10E	0.9800
C5—H5A	0.9900	C10'—H10F	0.9800
C5—H5B	0.9900	O2—H2A	0.85 (2)
C6—C7	1.525 (2)	O2—H2B	0.92 (2)
C7—C8'	1.535 (7)	O3—H3F	0.82 (2)
C7—C8	1.555 (7)	O3—H3G	0.86 (3)
			
C3—S1—C4	96.81 (8)	C6—C7—C8'	109.4 (4)
C6—N2—C2	117.26 (13)	N3—C7—C8	114.3 (5)
C6—N2—C5	125.82 (14)	C6—C7—C8	112.0 (4)
C2—N2—C5	116.91 (12)	C8'—C7—C8	5.8 (8)
C7—N3—H3A	118.0 (10)	N3—C7—H7A	108.3
C7—N3—H3B	113.3 (11)	C6—C7—H7A	108.1
H3A—N3—H3B	101.0 (15)	C8'—C7—H7A	114.1
C7—N3—H3C	107.4 (13)	C8—C7—H7A	108.3
H3A—N3—H3C	107.0 (17)	N3—C7—H7B	110.4
H3B—N3—H3C	109.8 (17)	C6—C7—H7B	110.2
N1—C1—C2	177.1 (2)	C8'—C7—H7B	110.1
N2—C2—C1	108.01 (12)	C8—C7—H7B	104.3
N2—C2—C3	112.16 (14)	H7A—C7—H7B	3.9
C1—C2—C3	112.35 (14)	C9—C8—C10	111.2 (6)
N2—C2—H2	108.1	C9—C8—C7	110.9 (7)
C1—C2—H2	108.1	C10—C8—C7	110.1 (6)
C3—C2—H2	108.1	C9—C8—H8	108.2
C2—C3—S1	113.17 (11)	C10—C8—H8	108.2
C2—C3—H3D	108.9	C7—C8—H8	108.2
S1—C3—H3D	108.9	C10'—C8'—C9'	112.1 (6)
C2—C3—H3E	108.9	C10'—C8'—C7	112.7 (6)
S1—C3—H3E	108.9	C9'—C8'—C7	114.5 (7)
H3D—C3—H3E	107.8	C10'—C8'—H8'	105.6
C5—C4—S1	111.44 (13)	C9'—C8'—H8'	105.6
C5—C4—H4A	109.3	C7—C8'—H8'	105.6
S1—C4—H4A	109.3	C8'—C9'—H9D	109.5
C5—C4—H4B	109.3	C8'—C9'—H9E	109.5
S1—C4—H4B	109.3	H9D—C9'—H9E	109.5
H4A—C4—H4B	108.0	C8'—C9'—H9F	109.5
N2—C5—C4	111.57 (13)	H9D—C9'—H9F	109.5
N2—C5—H5A	109.3	H9E—C9'—H9F	109.5
C4—C5—H5A	109.3	C8'—C10'—H10D	109.5
N2—C5—H5B	109.3	C8'—C10'—H10E	109.5
C4—C5—H5B	109.3	H10D—C10'—H10E	109.5
H5A—C5—H5B	108.0	C8'—C10'—H10F	109.5
O1—C6—N2	121.61 (15)	H10D—C10'—H10F	109.5
O1—C6—C7	119.60 (13)	H10E—C10'—H10F	109.5
N2—C6—C7	118.67 (13)	H2A—O2—H2B	97 (2)
N3—C7—C6	105.67 (11)	H3F—O3—H3G	103 (2)
N3—C7—C8'	111.0 (5)		
			
C6—N2—C2—C1	−114.30 (15)	N2—C6—C7—N3	162.20 (13)
C5—N2—C2—C1	66.80 (17)	O1—C6—C7—C8'	97.7 (6)
C6—N2—C2—C3	121.36 (15)	N2—C6—C7—C8'	−78.2 (6)
C5—N2—C2—C3	−57.54 (17)	O1—C6—C7—C8	103.3 (5)
N1—C1—C2—N2	49 (4)	N2—C6—C7—C8	−72.7 (5)
N1—C1—C2—C3	173 (4)	N3—C7—C8—C9	52.9 (9)
N2—C2—C3—S1	57.51 (16)	C6—C7—C8—C9	−67.3 (10)
C1—C2—C3—S1	−64.39 (17)	C8'—C7—C8—C9	−3(8)
C4—S1—C3—C2	−54.02 (14)	N3—C7—C8—C10	−70.6 (9)
C3—S1—C4—C5	56.05 (13)	C6—C7—C8—C10	169.3 (7)
C6—N2—C5—C4	−118.33 (16)	C8'—C7—C8—C10	−127 (9)
C2—N2—C5—C4	60.48 (18)	N3—C7—C8'—C10'	−61.6 (9)
S1—C4—C5—N2	−61.99 (17)	C6—C7—C8'—C10'	−177.9 (8)
C2—N2—C6—O1	−4.4 (2)	C8—C7—C8'—C10'	64 (8)
C5—N2—C6—O1	174.41 (15)	N3—C7—C8'—C9'	68.0 (10)
C2—N2—C6—C7	171.52 (12)	C6—C7—C8'—C9'	−48.2 (11)
C5—N2—C6—C7	−9.7 (2)	C8—C7—C8'—C9'	−166 (9)
O1—C6—C7—N3	−21.81 (18)		

### Hydrogen-bond geometry (Å, °)

**Table:**

*D*—H···*A*	*D*—H	H···*A*	*D*···*A*	*D*—H···*A*
N3—H3A···O2^i^	0.96 (2)	1.89 (2)	2.838 (2)	169.3 (15)
N3—H3B···Cl1	0.97 (2)	2.37 (2)	3.3178 (16)	165.3 (15)
N3—H3C···O1^ii^	0.807 (18)	2.232 (18)	2.7891 (17)	126.6 (17)
N3—H3C···Cl1^ii^	0.807 (18)	2.599 (19)	3.2732 (16)	142.0 (16)
O2—H2A···Cl1^i^	0.85 (2)	2.37 (3)	3.2176 (15)	171 (2)
O2—H2B···O3	0.92 (2)	1.81 (2)	2.7186 (19)	171 (2)
O3—H3F···Cl1	0.82 (2)	2.31 (2)	3.1332 (15)	179 (2)
O3—H3G···O2^i^	0.86 (3)	2.02 (3)	2.873 (2)	173 (2)

Symmetry codes: (i) −*x*+1, *y*+1/2, −*z*+1; (ii) −*x*, *y*+1/2, −*z*+1.
